# Impact of Artificial Intelligence on Regional Green Development under China’s Environmental Decentralization System—Based on Spatial Durbin Model and Threshold Effect

**DOI:** 10.3390/ijerph192214776

**Published:** 2022-11-10

**Authors:** Yuxin Fang, Hongjun Cao, Jihui Sun

**Affiliations:** School of Management, Ocean University of China, Qingdao 266100, China

**Keywords:** artificial intelligence (AI), green total factor productivity (GTFP), environmental decentralization, technological innovation, regional absorptive capacity, moderating effect, spatial Durbin model (SDM), threshold regression model

## Abstract

Artificial intelligence (AI) is the core technology of digital economy, which leads the transition to a sustainable economic growth approach under the Chinese-style environmentally decentralized system. In this paper, we first measured the green total factor productivity (GTFP) of 30 Chinese provinces from 2011 to 2020 using the super-efficiency slacks-based measure (SBM) model, analyzed the mechanism of the effect of AI on GTFP under the environmental decentralization regime, and secondly, empirically investigated the spatial evolution characteristics and the constraining effect of the impact of AI on GTFP using the spatial Durbin model (SDM) and the threshold regression model. The findings reveal: a U shape of the correlation of AI with GTFP; environmental decentralization acts as a positive moderator linking AI and GTFP; the Moran index demonstrates the spatial correlation of GTFP; under the constraint of technological innovation and regional absorptive capacity as threshold variables, the effect of AI over GTFP is U-shaped. This paper provides a useful reference for China to accelerate the formation of a digital-driven green economy development model.

## 1. Introduction

The work undertaken by Chung et al. (1997) was the initiative to incorporate undesirable outputs into the measurability of productivity for the first time [1], on which the notion of green total factor productivity (GTFP) was formulated. The current trend of global warming and resource constraint is becoming more and more serious, and the tension between Chinese economic progress, environmental conservation, as well as depletion of resources is becoming more and more prominent [2]. In October 2022, the 20th National Congress of the Communist Party of China reported that we should promote the high-end, intelligent, and green development of the manufacturing industry, promote green and low-carbon economic and social development, which is a key link to achieving high-quality development, improve the market-based allocation system of resource and environmental factors, and accelerate the research, development, and application of energy-saving and carbon-reducing advanced technologies. The access towards achieving sustainability of economic expansion lies in enhancing GTFP [3,4], which is a valid and comprehensive indicator of quality economic development. Artificial intelligence (AI) is often broadly described in terms of systems that accurately interpret exterior data, has the capability of learning from that data, in addition to using that acquisition to implement particular goals and missions by means of adaptive agility [5]. The Ministry of Science and Technology 2022 issued a “notice on supporting the construction of a new generation of artificial intelligence demonstration application scenarios” that requires giving full play to the role of artificial intelligence to empower economic and social development, and building a whole chain and process of artificial intelligence industry application ecology. The application of artificial intelligence in the chain makes it easier to diminish the overall influence over the environment by eliminating the need for excessive use of resources [6], and artificial intelligence drives the evolution of the economy from labor- and capital-intensive to technology-intensive [7], changing the way society and the economy, as a whole, operate [8]. Artificial intelligence can extract the value of production data, identify energy consumption bottlenecks, reduce production costs, and refine clean production management [9]. The greatest advantage of exploring the link between AI and GTFP is the combined impact of economic growth, energy efficiency, and the environment that can be derived [7].

With greenery having emerged as the foundation of China’s current high-level construction [10], the Chinese government leads the way towards the strategic deployment of environmental protection, proposing to establish a government-led, social organizational and publicly engaged multi-governance, source-prevention environmental governance system [11]. As a part of environmental governance system reform [12], the scientifically sensible allocation of administrative power of environment across central and local governments [13] serves as the systematic basis for improving the efficiency of green development, while environmental decentralization determines the structure and utilization effectiveness of investment on environment pollutant management [14], so environmental decentralization plays an important role in influences exerted by AI technology on GTFP.

Many studies have been conducted to test the implications of artificial intelligence as a powerful driver on green economic growth [15], green human resource management [6], technological innovation performance [8,16], and green finance [17]. Zhao et al. (2022) found a remarkable “U-shaped” contribution of AI to GTFP and found that increasing AI levels in resource-rich areas improved GTFP [18]. The research on AI and government environmental governance systems is mostly explored in terms of the application of AI technologies in environmental modernization [19,20,21], ensuring that emerging technologies such as AI are fully utilized by building new frameworks, policies, and governance [22]. Current scholarship on GTFP still focuses on policy evaluation and production drivers [23]. Artificial intelligence is an important way to alleviate the current pressure on resources and the environment [24]. Although current research has widely discussed AI, however, there is still a lack of investigation into the implications of AI upon regional greener development in the context of an environmental decentralization system with Chinese characteristics. We are endeavoring to navigate the mysteries of China’s peculiar environmental decentralization regime with respect to the connection with AI and GTFP.

This paper, therefore, addresses the following questions and provides a marginal contribution to research in related fields: what is the impact of AI on regional green development under a Chinese-style environmental decentralization system? Is there heterogeneity in the impact of AI, environmental decentralization, and GTFP across provinces? Is the GTFP spatially correlated and is there a spatial spillover effect of AI on the GTFP? Do technological innovation and regional absorptive capacity pose constraints on the process by which AI affects GTFP? The answers to the above questions in this study are of guiding significance for better ecological and economic co-wins in the digital economy. In the current environment of decentralization of environmental management affairs between the central government and local governments in China, studying the integration of artificial intelligence and green total factor productivity provides a power source to promote high-quality development and transformation and upgrading of China’s economy, and relying on the integration of the two can form a new pattern of green development.

The rest of this paper is organized as follows: Section 2 is a literature review, summarizing the current studies’ status and shortcomings; Section 3 explains the study methodology of this paper, including model setting, variable description, and sources of data; Section 4 is the interpretation of the empirical test outcomes; and Section 5 outlines and generalizes the findings of the paper, along with policy recommendations accordingly.

## 2. Literature Review

GTFP is a new total factor productivity accounting system based on the traditional green total factor productivity by further adding resource consumption to the input factor and incorporating non-desired output represented by pollutant emissions into the output factor [25], which is then calculated as GTFP. GTFP is the residual value of output growth after deducting the growth of various factor inputs and emissions [26]. Current research regarding GTFP concentrates mostly upon the influence elements and measurement models, and input and output indicators are consistent or similar across scholars’ regions [27,28]. Research on the factors influencing GTFP has focused on two perspectives [29], one exploring the impact of economic [30], technological [31], and resource [32] factors on the green development of national economies [33], and the other exploring the utility in shaping GTFP by government policies and legislative frames [34,35,36]. In terms of GTFP measurement, the dominant methodologies typically encountered are parametric, semi-parametric, and nonparametric processes [37]. To address the radial and directional bias in GTFP growth, the super slacks-based measure (SBM) model suggested by Tone [38] has become the most widespread tool in assessing green productivity growth [29].

Theories, methods, and techniques that help machines analyze, simulate, exploit, and explore human thought processes and behavior can be considered as artificial intelligence [39]. With the rise of Industry 4.0, the use of big data and artificial intelligence in creating value is becoming more and more widespread [40]. In their book *Data Science Applied to Sustainability Analysis*, Dunn and Balaprakash (2021) mention that data science and technology have become central to addressing sustainability challenges, and this role will only expand in the future [41]. In the era of Industry 4.0 artificial intelligence is important for fostering new competitive advantages, changing the energy consumption structure [42], promoting industrial transformation, as well as the advancement towards the middle and higher levels of the industrial value chain, which, in turn, exerts a significant influence upon sustaining the growth of China’s economy [43] and is one of the core factors for the development of GTFP. Artificial intelligence can influence GTFP in three ways: improving resource utilization in the production process, improving pollution treatment and pollution control, and fostering green industries and promoting green energy development [44].

However, there are few studies on the association with AI and GTFP. Bai and Sun (2021) studied the impact on total factor carbon productivity from the overall perspective of Internet development (including Internet+, big data, cloud computing, AI, etc.), which found that Internet development significantly contributed towards the enhancement of total factor carbon productivity [45]. Zhao’ s study (2022) is concerned with inclusive economic growth and focuses mainly on the analysis of the intrinsic drivers of inclusive economic growth [46]. Li and Song’s study (2022) constructs a green evaluation system based on three dimensions: economic, environmental, and social and investigates its intrinsic mechanisms [47]. Compared with the studies of Zhao (2022) and Li and Song (2022), this paper tends to study the extrinsic influences of GTFP. The study by Zhang et al. (2022) confirmed the U-shaped effect of AI on GTFP using a nonlinear dynamic panel regression model [16], which discussed the effect of regional resource heterogeneity but did not conduct an analysis of spatial spillover effects between regions. Lyu et al. (2022) confirmed the U-shaped relationship between both digital economy and GTFP, using a comprehensive indicator built from four dimensions of digital economy infrastructure, digital talent, digital application, and digital economy development environment to measure the digital economy [7]. In this paper, we refine the research object and select only one of the digital economy infrastructure indicators to explore whether AI will have a different impact on GTFP.

A substantial body of existing research concerns the mechanisms of the fiscal decentralization affecting GTFP [48,49]. Song et al. (2020) studied the effectiveness of decentralized fiscal authority on GTFP from a revenue perspective and an expenditure perspective, respectively [50]. Significant political heterogeneity occurs due to the existence of different political status and political treatment among regions, which leads to more resource allocation power for regions with higher political status [51]. However, fiscal decentralization is not equivalent with environment decentralization. Environmental decentralization refers to the rational distribution of environmental protection rights among all levels of government [52], with local governments choosing the type of environmental protection policy [53] that is appropriate to their local preferences and seeking the optimal allocation of environmental protection functions among all levels of government [54]. Fiscal decentralization emphasizes the attribution of economic rights in the central and local government, while environmental decentralization emphasizes the delineation of environmental regulation rights [55]. There is a lack of exploration into the implications of environmental decentralization over artificial intelligence.

To summarize, the current exploration of the connection among AI, environmental decentralization, and GTFP by domestic and foreign scholars is still insufficient, and the mode of functioning together with AI and GTFP remains ambiguous, ignoring the implications of China’s ambient policies on AI. Therefore, this article estimates the GTFP of 30 Chinese provinces from 2011 to 2020 and adds the interaction term of environmental decentralization and AI to probe the moderation function by environmental decentralization and tests the heterogeneity of the three districts in the eastern, central, and western regions of the country by adding an SDM to empirically study the spatial correlation and a threshold regression model to imperatively study the threshold constraint by technological innovation as well as regional absorptive capacity.

## 3. Methodology

### 3.1. Equation Setting

To verify the effectiveness in AI on GTFP, the following regression equation for panel data is developed:

(1) $${\mathrm{GTFP}}_{\mathrm{it}}{=\alpha }_{1}{\mathrm{AI}}_{\mathrm{it}}{+\alpha }_{2}X_{\mathrm{it}}{+\mu }_{i}{+\lambda }_{t}{+\varepsilon }_{\mathrm{it}}$$

(2) $${\mathrm{GTFP}}_{\mathrm{it}}{=\alpha }_{3}{\mathrm{AI}}_{\mathrm{it}}{+\alpha }_{4}{\mathrm{AI}\_\mathrm{sq}}_{\mathrm{it}}{+\alpha }_{5}X_{\mathrm{it}}{+\mu }_{i}{+\lambda }_{t}{+\varepsilon }_{\mathrm{it}}$$

In Equation (1), AI is the core explanatory variable, standing for artificial intelligence. GTFP is the explanatory variable and signifies GTFP. X is the control variable, i and t indicate the i-th region and t-th period, respectively, μ_i_ serves as an individual fixed effect, λ_t_ serves as a time fixed effect, and ε_it_ works as a random disturbance term. To verify the potential nonlinear effects of AI upon the presence of GTFP, Equation (2) introduces the squared term of AI. AI_sq is the square of artificial intelligence.

In order to further verify the moderating function ascribed to environmental decentralization over the correlation among AI and GTFP, this paper introduces the interaction terms of AI, squared AI, and environmental decentralization, respectively, centered on AI*ED and AI_sq*ED, and Equation (3) is constructed.

(3) $${\mathrm{GTFP}}_{\mathrm{it}}{=\beta }_{1}{\mathrm{AI}}_{\mathrm{it}}{+\beta }_{2}{\mathrm{AI}\_\mathrm{sq}}_{\mathrm{it}}{+\beta }_{3}{\mathrm{ED}}_{\mathrm{it}}{+\beta }_{4}{\mathrm{AI}}_{\mathrm{it}}{*\mathrm{ED}}_{\mathrm{it}}{+\beta }_{5}{\mathrm{AI}}_{{\mathrm{sq}}_{\mathrm{it}}}{*\mathrm{ED}}_{\mathrm{it}}{+\beta }_{6}X_{\mathrm{it}}{+\mu }_{i}{+\lambda }_{t}{+\varepsilon }_{\mathrm{it}}$$

In Equation (3), ED is the moderating variable, representing environment decentralization. AI*ED represents the interaction term between AI and ED, and AI_sq*ED represents the interaction term between AI_sq and ED.

The GTFP of a region may be subject not merely to the level of AI within the locality, but also to level of AI in its neighboring areas. Therefore, the following two spatial econometric equations are set up to test the spatial spillover effectiveness of AI and environmental decentralized regulatory mechanisms:

(4) $${\mathrm{GTFP}}_{\mathrm{it}}{=\rho \mathrm{WGTFP}}_{\mathrm{it}}{+\gamma }_{1}{\mathrm{WAI}}_{\mathrm{it}}{+\gamma }_{2}{\mathrm{WAI}\_\mathrm{sq}}_{\mathrm{it}}{+\gamma }_{3}{\mathrm{WX}}_{\mathrm{it}}{+\lambda }_{t}{+\varepsilon }_{\mathrm{it}}$$

(5) $${\mathrm{GTFP}}_{\mathrm{it}}{=\beta \mathrm{WGTFP}}_{\mathrm{it}}{+\delta }_{1}{\mathrm{WAI}}_{\mathrm{it}}{+\delta }_{2}{\mathrm{WAI}\_\mathrm{sq}}_{\mathrm{it}}{+\delta }_{3}{\mathrm{ED}}_{\mathrm{it}}{+\delta }_{4}{\mathrm{WAI}}_{\mathrm{it}}{*\mathrm{ED}}_{\mathrm{it}}{+\delta }_{5}$$

where W in Equations (4) and (5) is the spatial weight matrix.

Following Hansen’s (1999) study [56], we set technological innovation and regional absorptive capacity to be threshold variables to detect the effects generated by AI on GTFP at different levels of technological innovation and regional absorptive capacity, respectively, and construct a threshold regression, Equations (6) and (7).

(6) $${\mathrm{GTFP}}_{\mathrm{it}}{=\theta }_{0}{+\theta }_{1}{\mathrm{AI}}_{\mathrm{it}}\times I(\mathrm{TI}\leq {q)+\theta }_{2}{\mathrm{AI}}_{\mathrm{it}}{\times I(\mathrm{TI}>q)+\theta }_{3}X_{\mathrm{it}}{+\varepsilon }_{\mathrm{it}}$$

(7) $${\mathrm{GTFP}}_{\mathrm{it}}{=\theta }_{0}+\theta_{1}{\mathrm{AI}}_{\mathrm{it}}\times I(\mathrm{RAC}\leq q)+\theta_{2}{\mathrm{AI}}_{\mathrm{it}}\times I(\mathrm{RAC}>q)+\theta_{3}X_{\mathrm{it}}+\varepsilon_{\mathrm{it}}$$

where I() is the indicator function and q is the threshold value.

### 3.2. Variable Selection

Explained variables: green total factor productivity (GTFP). The study by Feng and Zhang (2017) measured GTFP using the weak directional distance function (W-DDF) model, strong directional distance function (S-DDF) model, and SBM model, respectively, and the results showed that the SBM model corresponds better to the true interpretation of GTFP [57]. Since the super-efficient SBM model evaluates the quiescent efficiency instead of GTFP, the Malmquist–Luenberger index derived from the super-efficient SBM model is chosen for computing the GTFP [58]. Motivated by the foregoing analysis, this article draws on the non-radial super-efficient SBM–Malmquist–Luenberger method commonly used by previous scholars [59,60], integrates energy expenditure and ambient contamination as part of the co-operative framework, and conducts the GTFP of 30 Chinese provinces (not including Tibet and Hong Kong, Macao, and Taiwan in view of data availability) from 2011 to 2020.

Assume that every province is treated like a decision-making unit (DMU) with a total of n DMUs. Each DMU has m input factors x_i_ (i = 1, 2, …, m) and produces s_1_ desired output y^d^ and s_2_ undesired output y^b^. The super-efficient SBM model measures the following equation [58,59,61]:

(8) $$\rho =\min\frac{\frac{1}{\;m}\sum_{i=1}^{m}\frac{{\bar{x}}_{i}}{x_{\mathrm{ik}}}}{\frac{1}{{\;s}_{1}+s_{2}}\left(\sum_{r=1}^{s_{1}}\frac{{\bar{y}}_{r}^{d}}{y_{r0}^{d}}+\sum_{j=1}^{s_{2}}\frac{{\bar{y}}_{j}^{b}}{y_{j0}^{b}}\right)}$$

(9) $$\{\begin{array}{c} x_{0}=X\lambda +S^{-},y_{0}^{d}=Y^{d}\lambda -S^{d},y_{0}^{b}=Y^{b}\lambda -S^{b}\\ \bar{x}\geq \sum_{j=1,\neq 0}^{n}\lambda_{j}x_{j}\\ {\bar{y}}^{d}\leq \sum_{j=1,\neq 0}^{n}\lambda_{j}y_{j}^{d}\\ {\bar{y}}^{b}\leq \sum_{j=1,\neq 0}^{n}\lambda_{j}y_{j}^{b}\\ \bar{x}\geq x_{0},{\bar{y}}^{d}\leq y_{0}^{d},{\bar{y}}^{b}\geq y_{0}^{b}\\ \lambda \geq 0,\sum_{j=1,\neq 0}^{n}\lambda_{j}=1,S^{-}\geq 0,S^{d}\geq 0,S^{b}\geq 0,{\bar{y}}^{d}\geq 0 \end{array}$$

The output indicators of GTFP include two parts: expected output and non-desired output; the input metrics comprise three parts: labor, capital, and energy inputs. Each specific indicator measurement method is shown in Table 1.

Explanatory variable: artificial intelligence (AI). As the new technological revolution, with the new generation of information technology as the core, unfolds globally, it has triggered a new round of industrial changes and the profound convergence on artificial intelligence, as well as industry [45], which will affect the regional GTFP. Modeling on Borland and Coelli (2017) [64], this study uses the logarithm of the annual social fixed asset investment across the information technology (IT), computer services, and software sectors in each province for the purpose of capturing the degree of regional AI development. Fixed asset investment is an indicator of constructing and acquiring fixed assets completed in some period of time manifested as currency, which has a decisive contribution to the formation, as well as advancement, in industries within a region [65], so the whole society fixed asset investment within the IT, computer services, and software sectors directly determines the extent of AI development within the area; hence, this study uses this indicator to represent AI. In this paper, the squaring parameter of AI is also included for verifying the possible nonlinearity of the association of AI with GTFP.

Moderating variable: environmental decentralization (ED). Most former investigations indirectly gauge environmental decentralization through the use of indicators, such as dummy variables, the proportion of locally autonomous laws, or fiscal decentralization [13,66], which cannot precisely embody the substance within Chinese-style environmental decentralization, while the allocation of staff in central and local environmental conservation systems can reflect the degree of environmental decentralization [14], so this paper draws on Zhang and Li (2022) [12] and Ran et al. (2020) [67] to use the staffing in both the central and the local environmental protective system as a measure of environmental power sharing.

Threshold variables: technological innovation and regional absorptive capacity. Technological innovation (TI) is recognized to be a crucial driver of the clean engine economy, since it facilitates the emergence of innovative markets and enhances the effectiveness of energy distribution while reducing pollutant emissions [68,69], as measured in this study using the logarithm of the number of applications received per 10,000 people for three domestic invention patents in China. Regional absorptive capacity (RAC) not only enhances the ability to identify, transform, and apply knowledge and technology in the region, but also promotes innovation capacity, which, in turn, promotes regional green development [70]. Research and development (R & D) expenditure is the core element of regional absorptive capacity [71], so this study uses a logarithmic measure of R&D internal funding expenditures of local sector-affiliated research and development institutions across all regions.

Control variable: industrial structure (IS): the second sector tends to be heavily dominated by energy-intensive industries that release considerable quantities of CO_2_, compared to the high output and negligible CO_2_ emissions of the trivial sector [72]; increasing the share ascribed to the tertiary sector throughout the overall manufacturing sector will incline the industrial structure to assume a more critical position in boosting economic performance and reducing pollutants [73], contributing towards enhancing Chinese environmental performance. The ratio of tertiary sector to secondary sector output is measured annually for each region in this paper. Foreign direct investment (FDI): the halo effect of FDI and technological change spillovers contribute towards the enhancement of ambient properties [74], so this article uses a logarithmic measure of the actual annual use of FDI. Government intervention (GOV): fiscal decentralization endows local governments a moderate extent to fiscal independence and promotes rational allocation of resources [4], but fiscal decentralization can lead to GDP-only local governments, generating negative environmental externalities [75], and thus acting as a disincentive to GTFP. In this paper, we use the annual fiscal budget revenue shared by regional GDP in each region to measure. Energy consumption structure (ECS): traditional fossil energy forms a major source of energy with respect to China’s economic development [76], and traditional fossil energy possesses a significant detrimental influence upon the environment, and its predominant energy consumption structure has a profound impact on GTFP. This paper measures the energy consumptive structure in terms of calculating the proportion of annual electricity usage in total energy utilization by region according to China’s General Rules for Calculating Comprehensive Energy Consumption GB/T 2589-2020. Population density (POP): population density of each region can reflect the degree of aggregation of regional labor factors, which brings scale effect to a certain extent and provides an engine for regional GTFP development. This article utilizes the number of people living on each unit area of land in each region each year for measurement. Description statistics for all variables are displayed in Table 2.

### 3.3. Data Source

The above raw materials are obtained from China Statistical Yearbook, China Environmental Yearbook, China Environmental Statistical Yearbook, China Business Yearbook, China Energy Statistical Yearbook, Social Statistical Bulletin of National Economic and Social Development, INFOBANK China Statistical Database, and the official website of the People’s Bank of China.

## 4. Empirical Results

### 4.1. Baseline Regression Analysis

The Hausman test was performed to ascertain the appropriate econometric model, and Prob > chi2 = 0.0035, indicating that the stationary-effects model becomes the optimal model; consequently, the fixed-effects model is used for the empirical investigation in this paper. Model 1 and Model 2 in Table 3 report the baseline regression analytical data.

**Figure 1:**
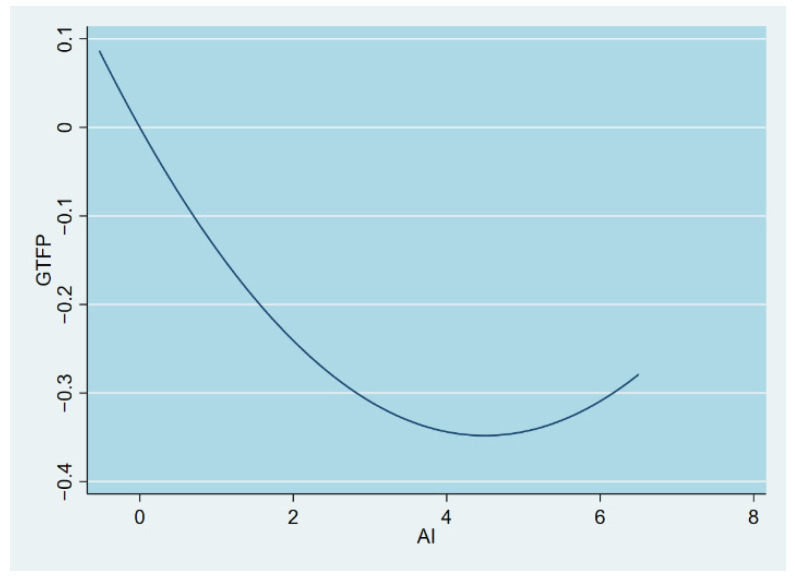
GTFP and AI relationship diagram.

The findings from Model 1 reveal that the coefficient of AI is statistically significantly negative, which demonstrates that AI can inhibit the progress of regional GTFP. The squared term of AI was added to Model 2 for probing the potential nonlinear influence existing in AI towards GTFP, and the coefficient of AI was found to be negative and the coefficient of AI squared was significantly positive, which illustrated that the influence of AI on GTFP is nonlinear and there may be a U-shaped correlation. This article uses utest [77] to corroborate this. utest’s results show that *p* > |t| = 0.006, verifying that the influence of AI over GTFP is indeed a U-shaped association. Figure 1 reports the U-shaped relationship between GTFP and AI. This is in agreement with the conclusions reached by Zhao et al. (2022) [18] and Lyu et al. (2022) [7]. However, the difference with the study of Zhao et al. (2022) lies in the choice of econometric model; the coefficients of AI and its quadratic term in the fixed effects model in the study of Zhao et al. (2022) are not significant but significant in the GMM model. The inflexion mark of the U-shape relationship is calculated to be 4.50, which implies that AI promotes GTFP when it is above 4.50 and acts as a disincentive when it is below 4.50. The possible reason for the above phenomenon is that the input cost of AI is high in the early stage and GTFP cannot compensate for the production cost, and, since there is a lag effect from capital investment to the utilization of new technology [78], AI plays a facilitating role for GTFP after the inflection point. Most provinces (except Shanxi, Liaoning, Hainan, Gansu, and Ningxia) exceed the inflection point value in 2020 and are in the right-hand phase of the U-shaped curve.

### 4.2. Analysis of Moderating Effects

**Figure 2:**
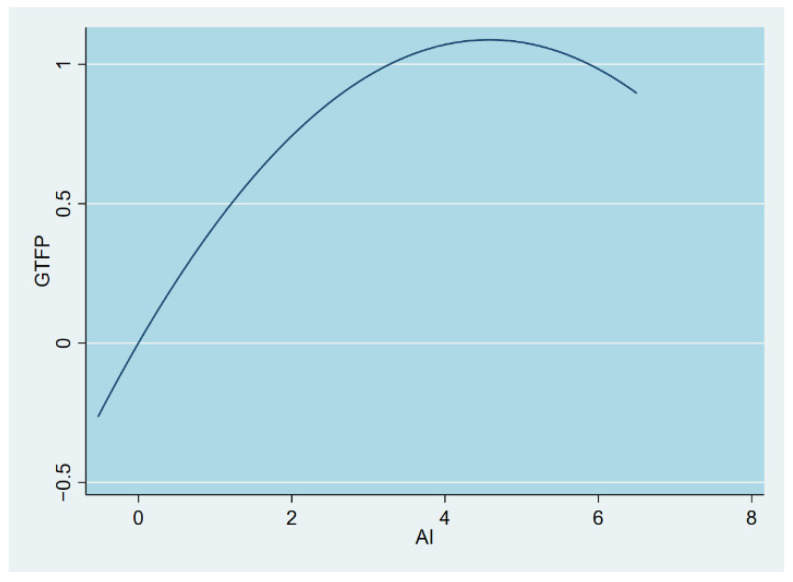
Graph of GTFP vs. AI (after adding moderation effect).

Lyu et al. (2022) conducted a mechanism test on the digital economy and GTFP and found that the impact of the digital economy on GTFP is mainly realized through factor market distortion and industrial structure upgrading [7]. In this paper, we choose another perspective of mechanism testing, i.e., we try to explore the moderating effect of environmental decentralization mechanism. Model 3 in Table 3 reports that the interaction term for environmental decentralization is significant with the 1% level, signifying that environmental decentralization has a moderating action in the influence of artificial intelligence on GTFP. The U-shaped relationship with moderating effects is further analyzed by drawing on Haans and Pieters (2016) [79] in order to clarify the moderating effects. The coefficient of the interactional term involving the square of AI and environmental decentralization in Model 3 is remarkably positive at the 1% level, indicating that environmental decentralization exerts a positive moderating effect within the U-shaped correlation between AI and GTFP, and the relationship between AI and GTFP tends to steepen, increasing severity of environmental decentralization. It shows that the environmental decentralization system allows local governments to have more abundant resources to govern the environment and increase the output of innovation, which leads to a closer relationship between AI and GTFP. The most interesting point is that the sign of the coefficients of both AI and its squared term becomes inverted after adding the moderating effect, and the curve shifts from a U shape to an inverted U shape under the moderation of environmental decentralization, as can be seen in Figure 2. This is further explained by the fact that the reverse U-shape correlation linking AI to GTFP is significantly strengthened in the case of high environmental decentralization. Referring to Haans and Pieters (2016) [79], the flip of the U-shape relativity may be accounted for in terms of the following mechanism: as the interacting term with respect to the strength of environmental decentralization and the intensity of AI relative to both is affected by the rise in technological dynamism, the U-shaped curve gradually bends toward the middle to the point where its curvature exceeds the curvature of the cost curve, so that the relationship changes from U-shaped to inverse U-shaped. After adding the moderating function of environmental decentralization, the inflexion mark of the inverse U-shape correlation among AI and GTFP becomes 4.58, and the inflexion mark of the inversed U-shape curve relocates to the right, which coincides with the tendency of the curve change in Figure 2.

### 4.3. Regional Heterogeneity Analysis

Models 4 to 9 in Table 4 report the differences in the effects of AI on GTFP and environmental regulation mechanisms in the eastern, central, and western regions of China, respectively, and the results reveal significant regional heterogeneity. China is a vast country, and there is a certain degree of uneven development in the eastern, central, and western regions; thus, the differences in the regression results in the models can be attributed to the local industry mix, development goals of each region [80], economic development level, and labor quality. The specific analysis in different regions is as follows. In Model 4, it is observed that the principal term of AI is statistically significantly negative and the semi-term is statistically significantly positive, together with its utest *p*-value of 0.034, so there exists a U-shape association among AI and GTFP at east. Model 5 becomes inverted U shape after adding the moderating force of environmental decentralization, which is the same trend as the national overall. The absolute values of the AI and AI-squared coefficients in Model 4 and the absolute values of the interaction term coefficients in Model 5 are larger compared to the national ones, indicating that the effect of AI upon GTFP, as well as the moderating function of environmental decentralization, are more significant in the east. The explanation could be that the influence by AI upon GTFP is more significant in the east as a developed region with faster development of technology and industrial revolution process [81], higher level of advanced industrial structure and local government environmental decentralization, and wide application of AI in emerging industries and tertiary industries. In Model 6, it can be seen that AI and its squared term coefficient are greater than the national level yet less than the level in the east, illustrating that the influences brought by AI on GTFP among the central area are superior to the national general level but still have a gap in comparison with the east. After adding the moderating effect of environmental decentralization across the Central Zone, the correlation between AI and GTFP remains U-shaped, which indicates that the degree of environmental decentralization among the central area does not yet support a flip in the U-shaped relationship. From Model 8, it can be seen that the coefficient of the primary term of AI is statistically significantly negative at the 10% level, while the quadratic term of AI is nonsignificant, indicating that AI has a simple linear relationship with GTFP and AI considerably suppresses the development of GTFP across the west. The interactive term between environmental decentralization and AI is added to Model 9, and the interaction term is significantly negative and the coefficient of AI is positive, implying that environmental decentralization in the western region acts as a negative moderator among AI and GTFP and, after moderating AI, plays a facilitating role to GTFP. The reason may be that, before regulation, AI and GTFP follow the cost hypothesis and the investment in AI does not achieve the effect of promoting higher GTFP and the costs outweigh the benefits. Meanwhile, after regulation, environmental decentralization promotes local governments to conduct R&D, following the Porter effect [82], producing a win–win situation.

### 4.4. Analysis of Spatial Effects

#### 4.4.1. Analysis of Spatial Correlation

Spatial econometric analysis presupposes the existence of spatial correlation [83] and, in this paper, the global Moran index is computed on the basis of the economic proximity weighing matrix [84] for the GTFP of 30 provinces from 2011 to 2020 to verify the spatial correlation. Table 5 demonstrates that the z-statistics of the global Moran index of GTFP from 2011 to 2020 all passed the significance test at the 1% level, and there are positive and negative global Moran indices, illustrating that GTFP exhibits a spatial positive correlation in some years and spatial negative correlation in some years, demonstrating that GTFP shows a spatial aggregation pattern throughout China.

**Figure 3:**
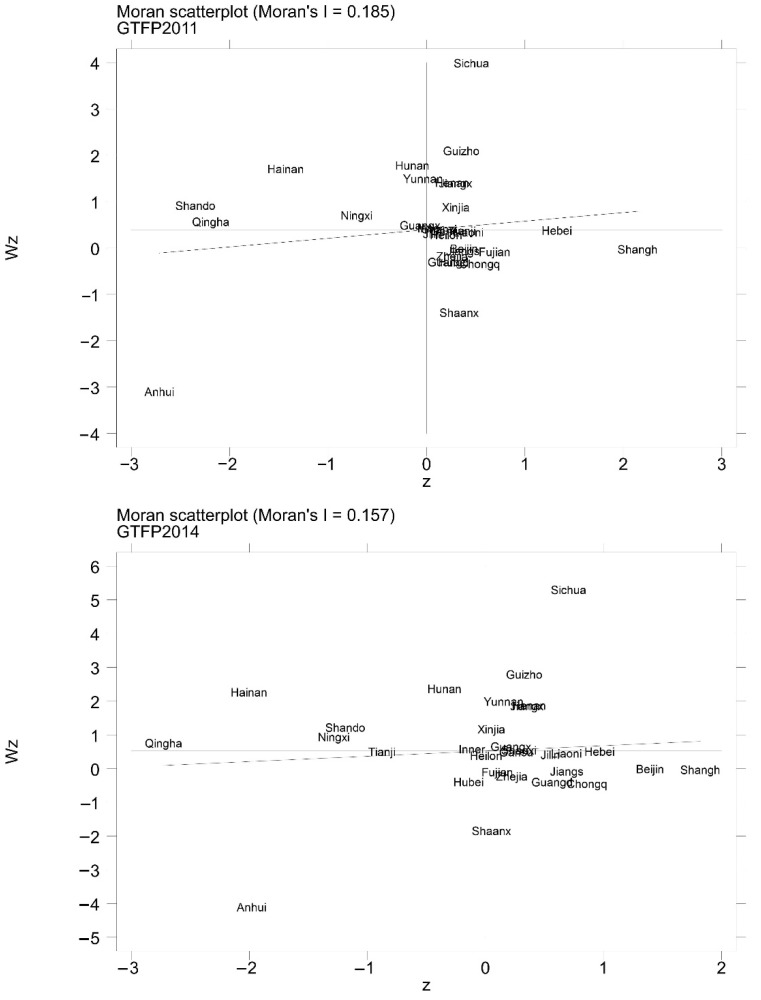

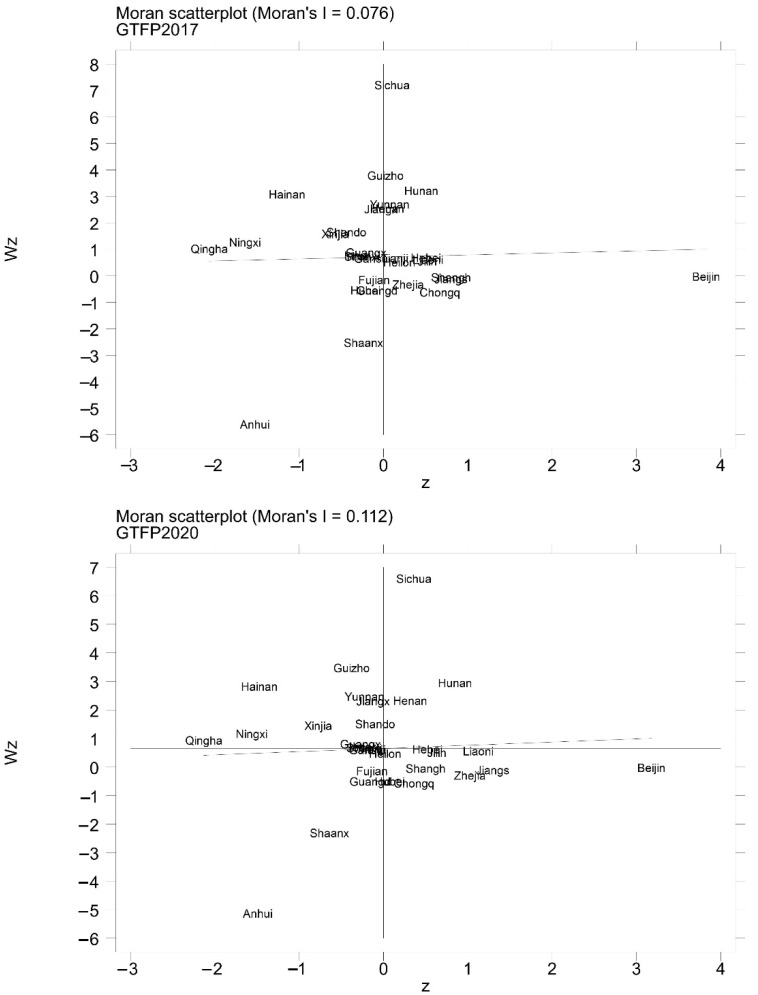
Moran scatter plot.

Based on the above analysis, local indicators of spatial association (LISA) [85] is further calculated and Moran scatter plots are drawn in order to explore local spatial correlation, i.e., spatial heterogeneity. In this paper, the data of 2011, 2014, 2017, and 2020 are selected and the Moran scatter plot is shown in Figure 3. As reflected in the figure, it is clear that the positions of the provinces basically do not change in the four years selected, which proves the stability of the spatial dependence of China’s GTFP. The GTFP shows an overall trend of shifting to the left during 2011–2020, demonstrating that the spatial dispersion of the GTFP converges to be obvious in 2011 and 2020. The four quadrants of the Moran scatter plot are high–high, low–high, low–low, and high–low aggregation, in that order [86], so it can be seen that the number of high–high and high–low aggregation provinces decreases, while the number of low–high and low–low aggregation provinces increases between 2011 and 2020, indicating a notable local spatial correlation in GTFP. The number of low factor productivity provinces increases and shows an increase in the gap between some low-GTFP provinces and neighboring provinces, as well as an increase in the similar aggregation effect in another part of low-GTFP provinces. Stably located in high–low agglomeration cities possessing a high level of GTFP and low level of GTFP in neighboring provinces are mainly Shanghai, Beijing, Jiangsu, Zhejiang, Chongqing, Hebei, etc. These areas have obvious advantages in terms of economic development level, advanced science and technology, and labor quality, so they can make their GTFP reach a higher level.

#### 4.4.2. Spatial Econometric Analysis

After the Moran index test mentioned above, this article proposes to introduce a spatial econometric model for the empirical analysis of spatial correlation. Through Hausman test, this study utilizes a spatial fixed effects model. For determining the optimal spatial econometric model, the Lagrange multiplier test, i.e., LM test [87], likelihood ratio, i.e., LR test, and Wald test are first conducted for the model spatial error model (SEM), spatial lag model (SAR), and spatial Durbin model (SDM), in turn, in this study. First, the LM test was performed. Table 6 shows that the Lagrange multipliers of both SEM and SAR were significant and could not significantly favor SEM or SAR, so the LR test and Wald test were undertaken. Table 6 reports that the *p*-values of both the LR test and the Wald test are significant, so the SDM does not deteriorate into SEM and SAR, and choosing of SDM is robust. Finally, to determine whether to choose the time fixed effect, individual fixed effect, or double fixed effect model, the LR test was performed again and the individual fixed effect could not reject the original hypothesis, while the time fixed effect refused the original assumption, so the time fixed effect was chosen.

Model 11 in Table 7 reports the baseline regression outcomes for SDM; Model 12, Model 13, and Model 14 report the decomposition findings regarding direct, indirect, and total effects; and Model 15 reports the spatial assessment findings of the environmental decentralized moderating influence. The R^2^ in Model 11 is 0.44 and the Log-likelihood is 111.82, indicating a good model fit and credibility. In Model 12 and Model 13, the direct effectiveness of the squared term of AI qualified the 1% significance test and the indirect effect was negative and insignificant, demonstrating that AI is advantageous in promoting the enhancement of GTFP within the region, while there is no significant spillover effect onto the peripheral areas. The interacting term of environmental decentralization and AI squared in Model 15 is not significant in the spatial weight matrix, indicating that environmental decentralization exerts a non-moderating function in the process of AI’s influence on GTFP.

#### 4.4.3. MAUP Test

**Figure 4:**
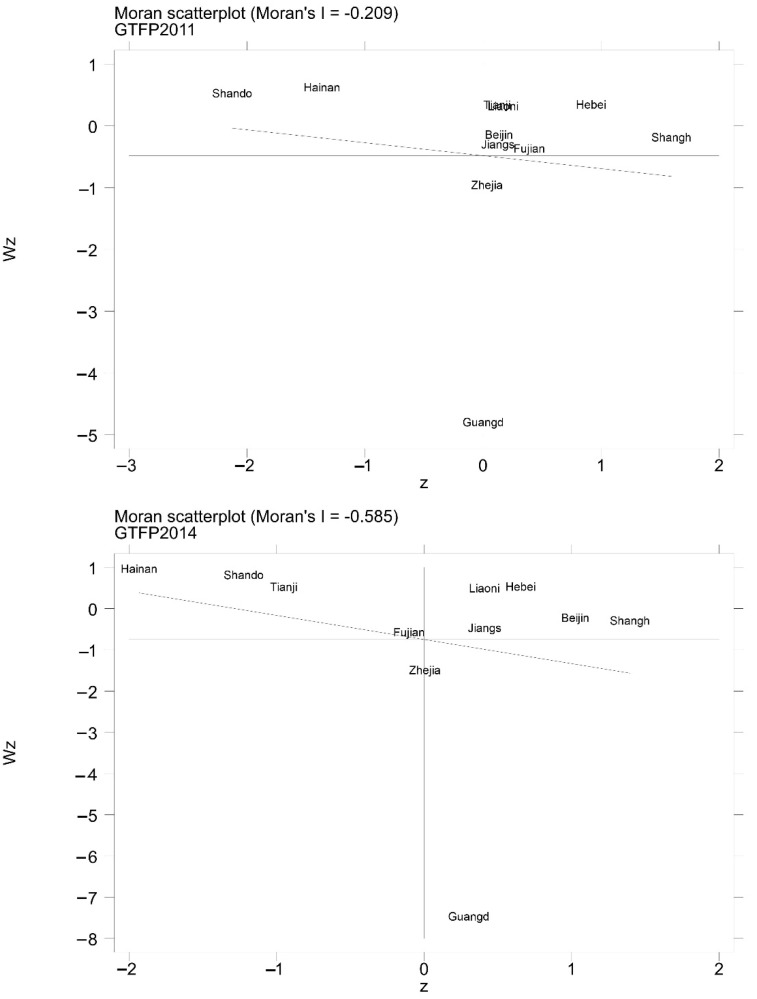

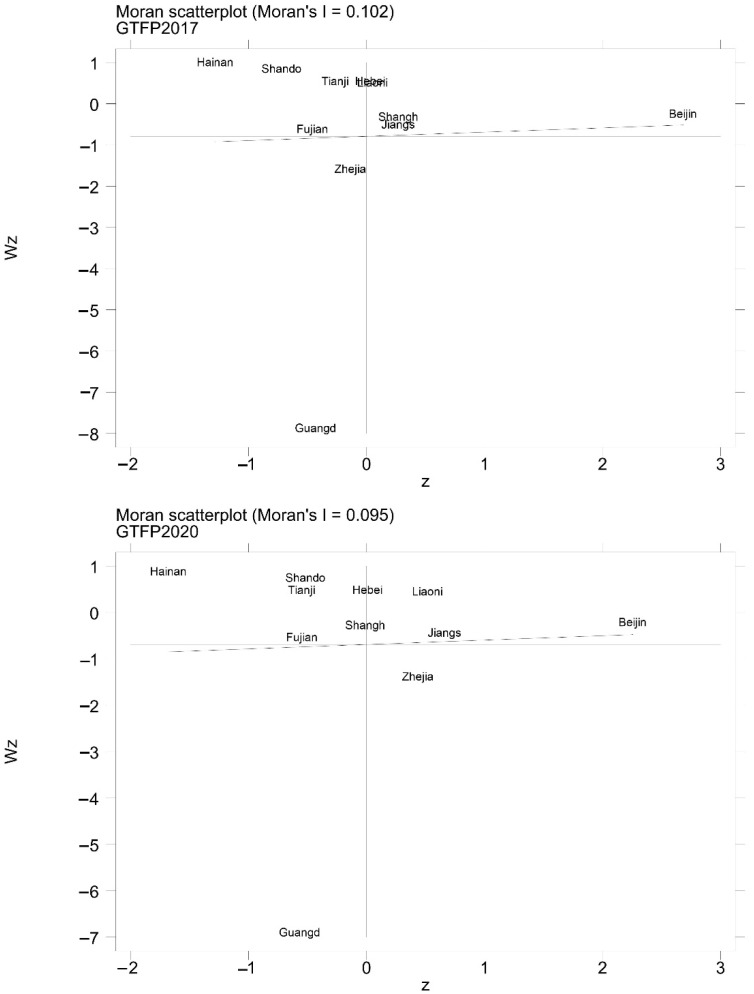
Moran scatter plot—based on MAUP.

The modifiable areal unit problem (MAUP) is a problem where the delineation of the area units of the study object may affect the results of the analysis, and a particular aggregation at a particular scale can produce results that are valid for that particular description [88,89]. The spatial effect is further explored in this paper by referring to the MAUP test used in the paper by Contreras (2022) [90]. Therefore, the entire eastern region was chosen for this paper to investigate whether different study area sizes would have an impact on the previous findings. First, the spatial autocorrelation test was conducted for the eastern region, and the global Moran index was significant (the following condition was satisfied from 2012 to 2020: *p* = 0.000). Next, the local Moran index test was performed and Figure 4, the Moran scatter plot, was plotted. Figure 4 reports the spatial distribution of GTFP for each province in the eastern region in 2011, 2014, 2017, and 2020, respectively. It can be seen from the figure that the position of the provinces in the eastern region in the quadrant has changed and the vast majority of the provinces are concentrated in the H-H region and the L-H region, and the overall trend of migration from the H-H region to the L-H region from 2011 to 2020 indicates that there is a trend of widening the GTFP gap between the provinces in the eastern region and a polarization phenomenon. Shandong and Hainan have been located in the second quadrant, but the position of Shandong Province has been shifting to the right, and the position of Hainan Province has not changed much. Tianjin and Fujian have slowed down the pace of development over the decade and the GTFP gap with surrounding provinces has increased.

Under the aforementioned condition of significant spatial correlation, this paper conducted a spatial econometric analysis using the spatial Durbin model. The results of the spatial regression are reported in Table 8. In Model 16, it can be seen that, under the economic distance weight matrix, the primary and secondary coefficients of AI are significantly positive and negative at the 1% level, respectively, so there is still a U-shaped relationship between AI and GTFP in space, and both the primary and secondary coefficients of AI are larger than those in Model 11. This indicates that the effect of AI on GTFP is greater in the eastern region, which is indeed consistent with the actual situation. At a finer spatial scale, labor and capital flows and trade are more important [91]. The eastern region has advantages in the level of technological development, the quality of human resources, and the level of advanced industrial structure, and AI is more widely used in the eastern region, so the enhancement effect of AI on the development of GTFP in the eastern region is greater. Models 17 to 19 report the decomposition effect of the spatial Durbin model measurement results, and the direct effect of AI on GTFP in the eastern region passes the test at the 1% level, indicating that there is no spatial spillover effect in the eastern region as well. The data of Model 20 show that the coefficients of AI primary term and square pass the test at the 1% level, respectively, and the AI primary term is positive and the squared term is negative, so the effect of AI on GTFP becomes inverted U-shaped under the moderation of environmental decentralization. This is the same direction of coefficients compared with Model 15, but the absolute values of the coefficients of the primary and quadratic terms of AI become larger in Model 20. A point of interest is that, under the economic distance weight matrix, the cross-product terms of the environmental decentralization and the AI primary and squared terms, respectively, are both significant at the 1% level, which indicates that, in the eastern region, AI can have an impact on GTFP only through the moderating effect of the environmental decentralization.

### 4.5. Robustness Test

For excluding the effect of extremes and outliers, a 1% tailoring of the data was performed [92], and the findings for the baseline regression, moderating effects, and SDM direct effects, indirect effects, and total effects are reported for Models 21 through 25 in Table 9, respectively. Model 26 to Model 30 in Table 10 is to add more control variables to test the robustness [93], adding outward foreign direct investment (OFDI); China makes full use of OFDI to learn and cite advanced technology at home and abroad and enhance our GTFP through reverse technology spillover from OFDI [94,95]. This study utilizes the ratio of gross exports and imports of merchandise over GDP, measured by regions by location of operating units. Except for a mild alteration concerning the absolute deviation of the coefficients, the symbols and the significances of the coefficients of AI and its square did not alter, and Model 22 and Model 27 report that environmental decentralization still positively moderates and that the association for AI and GTFP turns from U-shaped to inverted U-shaped, signifying that the aforementioned findings are robust.

### 4.6. Threshold Regression Analysis

This paper further employs a threshold regression model for exploring if the influence of AI over GTFP is constrained by the regional absorptive capacity of technological innovation. Table 11 reveals the values of the threshold regression test, where the F-statistic of technological innovation is statistically significant on the 1% level and regional absorptive capacity is significant at the 5% level, both of which satisfy the single-threshold test and reject the dual-threshold test, indicating the existence of one threshold each for technological innovation and regional absorptive capacity.

Table 12 presents the outcomes for estimating the thresholds in terms of technological innovation and regional absorptive capacity; from the table, it is evident that the threshold for technological innovation is 4.27 and the threshold for regional absorptive capacity is 12.15.

Table 13 presents the findings of the threshold regression effects and, since both technological innovation and regional absorptive capacity are single thresholds, both divide the effect of AI on GTFP into two intervals, respectively. When technological innovation is less than or equal to 4.27, the effect coefficient is −0.02, significant at the 5% level, as well as, when technological innovation exceeds 4.27, the effect coefficient is 0.5, significant at the 1% level. When the regional absorptive capacity is less than or equal to 12.15, its impact coefficient is −0.03, which adopts the significance test at the 5% level; when the regional absorptive capacity is greater than 12.15, its impact coefficient is 0.01, not passing the significance test. The coefficient of AI changes from negative to positive after crossing the two thresholds, respectively, indicating that the influence of AI onto GTFP is U-shaped before and after the threshold of technological innovation and regional absorptive capacity. The reason for this may be that productivity is low when the threshold is not crossed due to the crowding-out effect [96] and, after crossing the threshold with the advancement of territorial technological innovation along with regional absorptive capacity, the level of learning, absorption, and internalization of knowledge in the region is enhanced, promoting the development of AI and, thus, GTFP.

## 5. Conclusions and Policy Implications

The paper empirically examines the effect of AI on GTFP in 30 Chinese provinces under the environmental decentralization system from 2011 to 2020 using the super-efficient SBM model, spatial Durbin model, and threshold regression model. The following study findings were obtained: (1) the impact of AI on GTFP is first inhibited and then promoted in a U shape, with an inflection point of 4.50; (2) environmental decentralization serves as a positively moderating function among AI and GTFP, and the impact of AI on GTFP changes from a U shape towards an inverse U shape after moderating, and the shape of the curve becomes steeper and the inflection point shifts to the right; (3) the results of the regional heterogeneity test reveal that the influence by AI over GTFP in the east is U-shaped and, under the positive regulation of environmental decentralization, the influence of AI upon GTFP flips to an inverse U shape, which is the same trend as the national change; the influence of AI upon GTFP in the central region is U-shaped and still U-shaped under the positive regulation of environmental decentralization, and environmental decentralization strengthens the influence of AI over GTFP; the west region has a remarkable suppressive function of AI on GTFP and, after adding the moderating variable environmental decentralization, AI manifests a significant promoting action upon GTFP; (4) the Moran index suggests that there exists spatial correlation over GTFP, and the empirical findings of the SDM demonstrate that AI poses a direct effect on GTFP within the area, with no spatial spillover effect on the surrounding regions; (5) with technological innovation and regional absorptive capacity as the threshold variables, the influence of AI upon GTFP appears U-shaped under the respective constraints of both. 

Combining the foregoing study findings, this article proposes the ensuing policy insights. We can derive general considerations from the Chinese sample [97]. Firstly, the advantages of AI application are fully utilized to promote technological innovation. The empirical study in this paper finds that the impact of AI on GTFP shows a U shape and most provinces cross the inflection point and are at the stage of positive correlation between the two. Therefore, we attach great importance to the importance of AI on GTFP, take technological innovation as the core driving force, strengthen the construction of regional intelligent supporting facilities, accelerate the innovation and upgrading of AI-related technologies, guide the penetration of AI technologies into the value and industrial chains of production, circulation, configuration, integration, and sewage, and develop advanced pollution control and emission reduction technologies to achieve sustainable economic and social development. The second is to strengthen the government’s environmental governance policy guidance, appropriately increase the decentralization of environmental rights, give full play to the initiative of local governments, play to local advantages, and give AI a relaxed and supportive development environment. In view of the spatial correlation of GTFP, inter-regional co-operation should be strengthened, resource allocation should be optimized, and the full flow of factors between different regions should be realized, thus promoting the synergistic development of different regions. The third is to develop differentiated AI development strategies according to local conditions, dissolve regional barriers, and promote synergistic development in various regions. The eastern and central regions should continue to deepen reforms building on the existing foundation, enhance the diversification of industrial agglomeration, optimize industrial structure, and develop low-pollution industries. The western region should rely on its own resource advantages, learn advanced experience from the east and central parts, and innovate economic development models to create new economic expansion points from which to protect the ecological landscape. The fourth is to strengthen regional absorption capacity, promote inter-industry and inter-regional resource knowledge sharing, promote the formation of regions to produce knowledge spillover effects and agglomeration effects, take advantage of external opportunities, and focus on cultivating the ability to identify and absorb the resources, technologies, and talents into substantial benefits.

## Figures and Tables

**Table 1:** Specification of GTFP input–output data.

Indicator Type	Indicator Designation	Indicator Meaning	Measurement Approach
Output	Expected output	Real GDP per year by region	Deflated by the regional GDP index and converted to GDP in constant 2000 prices
	Non-desired output	Annual environmental pollution composite index by region	Entropy method [62]
Input	Labor input	Annual year-end figures for all employed persons by province	Total amount of employed persons in urban entities, private enterprises, and self-employment in all provinces at annual year-end
	Capital input	Actual physical capital stock per year by province	Perpetual Inventory Method [63]
	Energy input	Total annual energy consumption by region	Direct access to the China Statistical Yearbook

**Table 2:** Descriptive statistics.

Variable Type	Variable Symbol	Variable Meaning	Mean	Standard Deviation	Min.	Max.
Explained Variable	GTFP	Green Total Factor Productivity	0.95	0.23	0.33	2.12
Core Explanatory Variable	AI	Artificial Intelligence	4.76	0.99	−0.52	6.50
Adjustment Variable	ED	Environmental Decentralization	1.13	0.47	0.50	3.20
Threshold Variable	TI	Technology Innovation	2.33	1.08	0.07	4.65
	RAC	Regional Absorptive Capacity	10.84	1.00	7.56	13.29
Control Variable	IS	Industry Structure	1.14	0.66	0.50	5.17
	FDI	Foreign Direct Investment	5.49	1.66	−1.22	7.72
	GOV	Government Intervention	0.11	0.03	0.06	0.23
	ECS	Energy Consumption Structure	0.16	0.04	0.08	0.26
	POP	Population Density	463.50	690.50	7.80	3829.00

**Table 3:** Baseline regression analysis.

Variables	Model 1 GTFP	Model 2 GTFP	Model 3 GTFP
AI	−0.03 **	−0.016 ***	0.48 ***
	(−2.51)	(−4.56)	(3.49)
AI_sq		0.02 ***	−0.05 ***
		(3.94)	(−3.54)
ED			1.28 ***
			(4.14)
AI*ED			−0.63 ***
			(−4.76)
AI_sq*ED			0.07 ***
			(4.91)
IS	0.22 ***	0.21 ***	0.21 ***
	(6.40)	(6.36)	(6.42)
FDI	0.03 ***	0.02 ***	0.02 ***
	(2.36)	(1.20)	(1.35)
GOV	−2.93 ***	−2.87 ***	−2.21 ***
	(−5.06)	(−5.08)	(−3.87)
ECS	0.56	−0.26	−0.49
	(1.16)	(−0.50)	(−0.98)
POP	0.00 **	0.00 ***	0.00 **
	(2.80)	(2.71)	(2.24)
_cons	0.46 ***	0.89 ***	−0.37
	(2.88)	(4.68)	(−1.04)
N	300	300	300
R^2^	0.34	0.38	0.43
R^2^_a	0.25	0.29	0.35

Note: *t*-test statistics in parentheses, ** *p* < 0.05, *** *p* < 0.01.

**Table 4:** Regional heterogeneity analysis.

Variables	Model 4 GTFP East	Model 5 GTFP East	Model 6 GTFP Central	Model 7 GTFP Central	Model 8 GTFP West	Model 9 GTFP West
AI	−0.67 ***	1.17 ***	−0.40 **	−1.19 **	−0.05 *	0.08 ***
	(−3.68)	(2.01)	(−2.65)	(−2.21)	(−1.84)	(3.59)
AI_sq	0.06 ***	−0.13 ***	0.05 ***	0.14 **	0.00	
	(3.31)	(−2.27)	(3.30)	(2.56)	(0.24)	
ED		2.91 ***		−1.87		0.52 ***
		(2.72)		(−1.53)		(5.06)
AI*ED		−1.38 ***		0.86		−0.12 ***
		(−3.29)		(1.60)		(−5.8)
AI_sq*ED		0.15 ***		−0.10 *		
		(3.61)		(−1.76)		
IS	0.35 ***	0.35 *	0.05	0.07	0.12 **	0.12 ***
	(6.10)	(5.58)	(1.04)	(1.49)	(2.69)	(3.39)
FDI	0.12 **	0.11 *	0.01	−0.00	0.02	0.02
	(3.45)	(2.99)	(0.32)	(−0.18)	(1.44)	(1.33)
GOV	−4.61 ***	−3.95 ***	−3.61 ***	−4.25 ***	−2.98 ***	−1.90 ***
	(−4.14)	(−3.51)	(−3.39)	(−4.06)	(−5.34)	(−3.69)
ECS	2.04	2.51 **	0.76	1.45	−0.61	−0.68
	(1.66)	(2.28)	(0.67)	(1.19)	(−1.35)	(−1.81)
POP	0.00	−0.00	−0.01*	−0.01**	0.00	0.00
	(0.79)	(−0.33)	(−1.91)	(−0.47)	(0.40)	(1.04)
_cons	1.40 **	−2.34	3.43 ***	6.62 ***	1.17 ***	0.41
	(2.26)	(−1.53)	(3.89)	(3.90)	(3.91)	(1.41)
N	110	110	80	80	110	110
R2	0.63	0.73	0.51	0.58	0.41	0.58
R2_a	0.55	0.67	0.41	0.46	0.31	0.49

Note: *t*-test statistics in parentheses, * *p* < 0.1, ** *p* < 0.05, *** *p* < 0.01.

**Table 5:** Global Moran I test for GTFP in China, 2011–2020.

Variables	I	E(I)	sd(I)	z	*p*-Value *
GTFP2011	0.60	−0.03	0.04	15.95	0.00 ***
GTFP2012	−0.72	−0.03	0.04	−15.49	0.00 ***
GTFP2013	3.09	−0.03	0.05	69.55	0.00 ***
GTFP2014	1.04	−0.03	0.04	25.05	0.00 ***
GTFP2015	0.27	−0.03	0.04	7.01	0.00 ***
GTFP2016	−2.62	−0.03	0.04	−59.03	0.00 ***
GTFP2017	2.42	−0.03	0.02	111.34	0.00 ***
GTFP2018	1.85	−0.03	0.04	45.87	0.00 ***
GTFP2019	−1.94	−0.03	0.04	−45.79	0.00 ***
GTFP2020	−2.05	−0.03	0.04	−52.13	0.00 ***

Note: * *p* < 0.1, *** *p* < 0.01.

**Table 6:** Results of the basic test of the spatial econometric model.

Test		Statistic	*p*-Value
LM test	LM-SEM	11.99	0.00 ***
	Robust LM-SEM	6.44	0.02 **
	LM-SAR	6.98	0.01 **
	Robust LM-SAR	1.43	0.29
Hausman test			0.05 *
LR test	SEM nested in SDM	15.64	0.03 **
	SAR nested in SDM	17.81	0.01 **
Wald test	SDM-Error	32.21	0.00 ***
	SDM-Lag	27.64	0.00 ***
LR test	Ind nested in both	8.54	0.58
	Time nested in both	360.48	0.00 ***

Note: * *p* < 0.1, ** *p* < 0.05, *** *p* < 0.01.

**Table 7:** Spatial basis regression results.

Variables	Model 11 SDM	Model 12 SDM Direct	Model 13 SDM Indirect	Model 14 SDM Total	Model 15 SDM Moderating
AI	−0.07	−0.07	0.00	−0.07	0.31
	(0.05)	(0.05)	(0.23)	(0.23)	(0.20)
AI_sq	0.01 **	0.01 **	0.00	0.01	−0.04 **
	(0.01)	(0.01)	(0.04)	(0.04)	(0.02)
ED					0.51
					(0.43)
AI*ED					−0.38 **
					(0.19)
AI_sq*ED					0.06 ***
					(0.02)
IS	0.15 ***	0.15 ***	0.09	0.25	0.09 ***
	(0.02)	(0.02)	(0.25)	(0.25)	(0.03)
FDI	0.04 ***	0.04 ***	−0.01	0.03	0.04 ***
	(0.01)	(0.01)	(0.09)	(0.09)	(0.01)
GOV	−1.03 *	−1.07 **	1.47	0.40	−0.33
	(0.55)	(0.53)	(4.07)	(4.11)	(0.56)
ECS	−1.18 ***	−1.14 ***	0.75	−0.39	−1.08 ***
	(0.33)	(0.35)	(8.27)	(8.31)	(0.33)
POP	0.00 *	0.00	−0.00	−0.00	0.00
	(0.00)	(0.00)	(0.00)	(0.00)	(0.00)
W*AI	0.02				0.42
	(0.22)				(2.00)
W*AI_sq	−0.00				0.06
	(0.04)				(0.31)
W*ED					4.97
					(23.35)
W*(AI*ED)					−1.02
					(4.89)
W*(AI_sq*ED)					0.04
					(0.19)
W*IS	0.06				0.88
	(0.22)				(4.06)
W*FDI	−0.02				0.32
	(0.09)				(1.69)
W*GOV	1.04				−9.35
	(3.60)				(44.87)
W*ECS	1.34				−23.53
	(7.56)				(118.70)
W*POP	−0.00				0.00
	(0.00)				(0.02)
sigma2_e	0.03 ***				0.03 ***
	(0.00)				(0.00)
Log-likelihood	111.82				125.09
R^2^	0.44				0.36

Note: standard errors are in parentheses, * *p* < 0.1, ** *p* < 0.05, *** *p* < 0.01.

**Table 8:** Spatial basis regression results—based on MAUP.

Variables	Model 16 SDM	Model 17 SDM Direct	Model 18 SDM Indirect	Model 19 SDM Total	Model 20 SDM Moderating
AI	−0.68 ***	−0.69 ***	0.39	−0.31	1.37 ***
	(0.16)	(0.17)	(0.66)	(0.65)	(0.52)
AI_sq	0.06 ***	0.06 ***	−0.04	0.02	−0.15 ***
	(0.02)	(0.02)	(0.08)	(0.08)	(0.05)
ED					3.09 ***
					(0.92)
AI*ED					−1.46 ***
					(0.37)
AI_sq*ED					0.15 ***
					(0.04)
IS	0.32 ***	0.32 ***	0.07	0.39	0.31 ***
	(0.07)	(0.07)	(0.27)	(0.26)	(0.08)
FDI	0.22 ***	0.22 ***	0.08	0.29 **	0.16 ***
	(0.04)	(0.04)	(0.12)	(0.12)	(0.04)
GOV	−5.32 ***	−5.34 **	2.46	−2.88	−4.45 ***
	(1.26)	(1.23)	(4.14)	(4.24)	(1.24)
ECS	3.12 **	3.39 ***	−2.74	−0.65	3.93 ***
	(1.52)	(1.56)	(10.49)	(10.41)	(1.37)
POP	−0.00	−0.00	0.00	0.00	0.00
	(0.00)	(0.00)	(0.00)	(0.00)	(0.00)
W*AI	0.38				13.97 ***
	(0.74)				(4.74)
W*AI_sq	−0.05				−1.40 ***
	(0.09)				(0.50)
W*ED					31.44 ***
					(8.90)
W*(AI*ED)					−13.93 ***
					(3.86)
W*(AI_sq*ED)					1.51 ***
					(0.40)
W*IS	0.13				−4.48 ***
	(0.31)				(1.03)
W*FDI	0.12				−3.01 ***
	(0.14)				(0.61)
W*GOV	1.49				40.64 ***
	(4.55)				(0)
W*ECS	−3.82				2.81
	(11.35)				(22.56)
W*POP	0.00				−0.07 **
	(0.00)				(0.03)
sigma2_e	0.01 ***				0.03 ***
	(0.00)				(0.00)
Log-likelihood	107.27				124.86
R^2^	0.01				0.00

Note: standard errors are in parentheses, ** *p* < 0.05, *** *p* < 0.01.

**Table 9:** Robustness test results—tailoring.

Variables	Model 21 GTFP	Model 22 GTFP	Model 23 SDM Direct	Model 24 SDM Indirect	Model 25 SDM Total
AI	−0.26 ***	0.37 ***	−0.13 ***	0.03	−0.11
	(−6.32)	(2.88)	(0.06)	(0.28)	(0.29)
AI_sq	0.03 ***	−0.04 ***	0.02 ***	−0.00	0.02
	(5.96)	(−2.85)	(0.01)	(0.04)	(0.04)
ED		1.29 ***			
		(4.50)			
AI*ED		−0.63 ***			
		(−5.17)			
AI_sq*ED		0.07 ***			
		(5.35)			
IS	0.17 ***	0.18 ***	0.13 ***	0.14	0.27
	(5.20)	(5.73)	(0.02)	(0.39)	(0.39)
FDI	0.01	0.01	0.04 ***	−0.02	0.02
	(0.62)	(0.84)	(0.01)	(0.08)	(0.08)
GOV	−2.86 ***	−2.19 ***	−0.89 *	0.79	−0.10
	(−5.48)	(−4.20)	(0.51)	(5.07)	(5.12)
ECS	−0.41	−0.69	−1.37 **	0.09	−1.28
	(−0.85)	(−1.49)	(0.34)	(8.32)	(8.36)
POP	0.00 ***	0.00 **	0.00	−0.00	−0.00
	(3.13)	(2.49)	(0.00)	(0.00)	(0.00)
_cons	1.17 ***	−0.07			
	(6.17)	(−0.20)			
N	300	300	300	300	300
R^2^	0.39	0.45	0.43	0.43	0.43
R^2^_a	0.30	0.37			

Note: Model 21 and Model 22 have *t*-test statistics in parentheses, and Model 23, Model 24, and Model 25 have standard errors in parentheses, * *p* < 0.1, ** *p* < 0.05, *** *p* < 0.01.

**Table 10:** Robustness test results—additional control variables.

Variables	Model 26 GTFP	Model 27 GTFP	Model 28 SDM Direct	Model 29 SDM Indirect	Model 30 SDM Total
AI	−0.15 ***	0.44 ***	−0.07	0.10	0.031
	(−4.39)	(3.21)	(0.05)	(0.32)	(0.32)
AI_sq	0.016 ***	−0.05 ***	0.01 **	−0.02	−0.01
	(3.72)	(−3.23)	(0.01)	(0.06)	(0.06)
ED		1.18 ***			
		(3.84)			
AI*ED		−0.58 ***			
		(−4.41)			
AI_sq*ED		0.06 ***			
		(4.48)			
IS	0.19 ***	0.20 ***	0.15 ***	0.02	0.18
	(5.56)	(5.94)	(0.02)	(0.31)	(0.32)
FDI	0.01	0.02	0.04 ***	−0.03	0.01
	(1.01)	(1.32)	(0.01)	(0.10)	(0.10)
GOV	−3.14 ***	−2.52 ***	−1.07 **	−0.31	−1.38
	(−5.59)	(−4.38)	(0.54)	(5.17)	(5.25)
ECS	−0.11	−0.38	−1.09 ***	4.97	3.87
	(−0.22)	(−0.77)	(0.41)	(11.82)	(11.86)
POP	0.00	0.00	0.00	−0.00	−0.00
	(1.55)	(1.30)	(0.00)	(0.00)	(0.00)
OFDI	−0.21 ***	−0.18 ***	−0.01		0.12
	(−3.10)	(−2.67)	(0.07)		(0.37)
_cons	1.15 ***	−0.06			
	(5.62)	(−0.16)			
N	300	300	300	300	300
R^2^	0.40	0.45	0.46	0.46	0.46
R^2^_a	0.31	0.36			

Note: Model 26 and Model 27 have *t*-test statistics in parentheses, and Model 28, Model 29, and Model 30 have standard errors in parentheses, ** *p* < 0.05, *** *p* < 0.01.

**Table 11:** Threshold regression test.

Threshold Variables	Number of Thresholds	F-Statistic	*p*-Value	Threshold Value		
				1%	5%	10%
TI	1	107.12 ***	0.00	35.30	27.53	22.56
	2	15.98	0.25	226.22	171.77	124.45
RAC	1	47.40 **	0.01	47.53	33.12	27.86
	2	23.93	0.15	54.73	36.35	27.64

Note: ** *p* < 0.05, *** *p* < 0.01.

**Table 12:** Estimation results of threshold values.

Threshold Variables	Number of Thresholds	Estimated Values	Confidence Intervals
TI	Single Threshold	4.27	[4.25, 4.31]
RAC	Single Threshold	12.15	[12.08, 12.22]

**Table 13:** Threshold panel effects.

Threshold Variables	Model 28 TI	Model 29 RAC
IFI (TI ≤ 4.27)	−0.02 **	
	(−2.21)	
IFI (TI > 4.27)	0.05 ***	
	(3.74)	
IFI (RAC ≤ 12.15)		−0.03 **
		(0.02)
IFI (RAC > 12.15)		0.01
		(0.62)
IS	0.17 ***	0.20 ***
	(5.92)	(6.37)
FDI	0.02 *	0.03 **
	(1.74)	(2.17)
GOV	−2.57 ***	−2.64 ***
	(−5.17)	(−4.85)
ECS	0.26	−0.02
	(0.62)	(−0.05)
POP	0.00 *	0.00 *
	(1.96)	(1.91)

Note: *t*-test statistics in parentheses, * *p* < 0.1, ** *p* < 0.05, *** *p* < 0.01.

## Data Availability

The data presented in this study are available on request from the corresponding author.

## Abbreviations

GTFP	Green Total Factor Productivity
SBM	Slacks-based Measure
SDM	Spatial Durbin model
AI	Artificial Intelligence
ED	Environmental Decentralization
W-DDF	Weak Directional Distance Function
S-DDF	Strong Directional Distance Function
DMU	Decision Making Unit
IT	Information Technology
TI	Technological Innovation
RAC	Regional Absorptive Capacity
R&D	Research and Development
IS	Industrial Structure
FDI	Foreign Direct Investment
GOV	Government Intervention
ECS	Energy Consumption Structure
POP	Population Density
SEM	Spatial Error Model
SAR	Spatial Lag Model
MAUP	Modifiable Areal Unit Problem
OFDI	Outward Foreign Direct Investment
